# Prediction and Explanation in a Postmodern World

**DOI:** 10.3389/fpsyg.2020.597706

**Published:** 2020-12-01

**Authors:** Joachim I. Krueger

**Affiliations:** Department of Cognitive, Linguistic & Psychological Sciences, Brown University, Providence, RI, United States

**Keywords:** prediction, explanation, experimentation, causal analysis, postmodernism

## Abstract

The experimental research paradigm lies at the core of empirical psychology. New data analytical and computational tools continually enrich its methodological arsenal, while the paradigm’s mission remains the testing of theoretical predictions and causal explanations. Predictions regarding experimental results necessarily point to the future. Once the data are collected, the causal inferences refer to a hypothesis now lying in the past. The experimental paradigm is not designed to permit strong inferences about particular incidents that occurred before predictions were made. In contrast, historical research and scholarship in other humanities focus on this backward direction of inference. The disconnect between forward-looking experimental psychology and backward-looking historical (i.e., narrative) psychology is a challenge in the postmodern era, which can be addressed. To illustrate this possibility, I discuss three historical case studies in light of theory and research in contemporary psychology.

## Introduction

“Wo der Brotgelehrte trennt, vereinigt der philosophische Geist.”

[Where the ordinary scholar divides, the philosophical spirit unites.]

– Friedrich Schiller, May 26, 1789, in his first lecture as chair of history at the University of Jena

Friedrich Schiller did not shy away from lofty goals. Envisioning a universal theory of history, he surely realized that his listeners were guided by more modest aspirations. Yet, lofty goals are useful, and often needed, because they appeal to our better ideals, even if the odds of reaching them are long. Many scientists and scholars today might agree that everything is ultimately connected. The *Kosmos*, as von Humboldt (1845–1858) taught, is one. In practice, however, it is difficult to work from a universal point of view, and the academy has found it expedient to create distinctive administrative units where researchers can ask narrow questions they find tractable given the theories and methods available in their fields. Administrators periodically affirm the need for interdisciplinary or translational research, and occasionally they provide funds to support it. Such initiatives are useful as they guard against a descent into a world in which members of different academic tribes no longer understand one another.

The descent into tribalism may be more threat than reality, but the differences in methods and modes of thinking among disciplines are striking. The challenge remains to find answers to the question of what different disciplines can offer one another and whether these gifts can be used to good effect. The research topic presented by *Frontiers in Psychology*, to which this article seeks to make a contribution, asks about “modern” and “postmodern” approaches, and how their differences might be overcome. Taking the terms “modern” and “postmodern” as they are commonly understood, the prospect of a full reconciliation seems remote (Bereiter, 1994). Postmodernism, as it emerged from French *théorie* sees itself as a revolution, with its *raison d’être* being the rejection of modernism (Pluckrose and Lindsay, 2020). Modernism, and the Enlightenment from which is sprang, is a necessary condition for postmodernism. “If the enlightenment did not exist, postmodernists would have had to invent it” (Schmidt, 2002, p. 432). The mutual distrust runs deep. Much like the surrealists did not wish to compromise with the realists, or the Copernicans did not wish to “split the difference” with the Ptolemaians,^1^ so postmodernists appear to have no use for a middle ground between their own views and those of the modernists. A key demand of postmodernism is the co-existence of multiple epistemologies and the rejection of any kind of value-based ranking (Feyerabend, 1975). As this demand amounts to a rejection of modernist “science” as a privileged paradigm (Adams St. Pierre, 2002), it is hard to see what a compromise might look like. Modernists for their part maintain that science is not just another epistemology, on a par, as Feyerabend would have it, with astrology or voodoo. Science does not reduce to scientism, as Hutchinson (2011) claimed it does. Science provides the tools to study the validity of astrology, voodoo, and intercessory prayer, whereas the inverse effort cannot get off the ground. The most penetrating criticisms of scientific methods come from within the scientific community itself, and they help to improve the enterprise.

## Preview

In this article, I accept the general legitimacy of the modernist conception of science while exploring questions touching on postmodern sensibilities. Specifically, I pose two questions that I consider to be tractable with standard epistemological tools. The first question, which is theoretical, is how the concepts of prediction and explanation as construed in the conventional hypothesis-testing experimental paradigm relate to the concept of explanation as seen in historical accounts of events that happened in the past and outside the laboratory, or “in the wild.” Taking a Bayesian perspective (Mandel, 2014), I frame this issue as a question of “reverse inference” (Krueger, 2017b). After reviewing the theoretical tools, I present a stylized thought experiment to illustrate the probabilistic association between forward and reverse inferences.

The second question, which is practical in nature, is whether theory and evidence-based psychological reconstructions of individuals’ past decisions or behaviors can enrich historical scholarship. To explore the potential contributions of psychological reconstruction, I present three case studies. The first case features Philipp von Hutten, a historical person; the second involves Gonzalo Guerrero, a man suspended between history and legend; the third involves Robinson Crusoe, a figure familiar from literature. All three men found themselves in challenging circumstances demanding life-and-death decisions. With regard to Hutten and Gonzalo, I ask whether experimental psychology can help explain some of their critical choices. For the case of Crusoe, I introduce theoretical tools provided by a heterodox branch of game theory in order to reconstruct the interpersonal power dynamics between him and Friday. There is a limited tradition of historical case studies guided by theory and evidence. Simonton (1998), for example, pioneered and refined historiometric analyses of individuals of great creative or productive achievement. Dörner and Güss (2011) described, analyzed, and evaluated Hitler’s rigid pattern of strategic decision-making in light of psychological theories of cognition and personality. These efforts fall within the purview of “differential psychology,” yielding inferences that are only loosely tied to theories of psychological processes. The present article seeks to explore possible contributions of experimental psychology.

To conclude this article, I revisit three conceptual issues, which, if resolved, can shed light on the linkages between prediction and explanation, and, by extension, the linkages between modern and postmodern psychology. I ask whether any differences exist between factual and fictional behavior that affect the tasks of prediction and explanation. I then ask in what way causal accounts go beyond mere category judgments. Finally, I ask whether outcome biases affect both prediction and explanation.

## Experimentation, Prediction, and Explanation

*“Most researchers are aware that randomized experiments are considered the ‘gold standard’ for causal inference.”* – Rohrer (2018, p. 27)

Academic psychology is modern in the sense that one of its core goals is to uncover general laws governing mind and behavior, and in that its principal instrument is the experiment (Wundt, 1874; Woodworth and Schlosberg, 1954). With the search for laws, and theoretical explanations of these laws, psychological science aims to attain a fuller understanding of the nature of mental life (Popper, 1962; Meehl, 1978). In the modernist understanding, natural laws are there to be discovered, described, and deployed. At the vanishing, or “omega,” point, science would reveal “how the mind works” and “where behavior comes from.” At that limit, there would be a comprehensive ability to predict and explain mental states and behavior. This modernist understanding is rather mechanistic and deterministic, and it may seem outdated.^2^ Postmodernism, much like quantum physics (Schrödinger, 1930), questions the validity of this paradigm, but it is not evident that postmodernism can ground itself in quantum theory. Nor that it would want to. To a committed postmodernist, Schrödinger’s equations are just another story. However, most modernist scientists recognize the limits of determinism and the irreducibility of deep uncertainty. Still, the modernist premise that human experience is in large part comprehensible and lawful implies that experimental research is a powerful, if probabilistic, way to attain some “explanation through prediction.”

Theories are prediction machines, and experiments are their testing grounds (see Hempel and Oppenheim, 1948; Cummins, 2000; Grosz et al., 2020;, for critical discussions).^3^ In the experimental context, theory and testing look to the future. Experimenters might well assert that an intervention “explains” a certain amount of the variance in the data, and that prediction proper is limited to the domain of machine learning, computational modeling (Yarkoni and Westfall, 2017) and mind-free behaviorism (Skinner, 1981). The view I take here is that experiments test theoretically grounded explanations (Deutsch, 2016; Holtz and Monnerjahn, 2017). Predictions, if corroborated by the data, can aid explanation. The data convey information about the hypothesis that predicted them, a hypothesis that now lies in the past (the current preference for pre-registration demands that it does). Inferences from the data back to the hypothesis, that is, to the statement that predicted the data, are “reverse,” and they are fraught with uncertainty (Krueger and Heck, 2017, 2018).

Many people, when first introduced to the science of psychology, declare that they too have a keen interest in understanding what happened. “I am always curious,” they say, “why people do what they do.” As this desire to know springs from a natural epistemic instinct, it can be unsettling to learn that experimental psychologists have little to say about this question. Their inability to explain a particular behavior better than the folk themselves comes as a painful realization. In this article, I intend to take a small step toward a clarification of the nature of the disconnect between prediction and explanation by providing examples showing that findings obtained in the context of prediction can improve explanations of past events.

Postmodernism, at least in its early deconstructionist stage, rejected the idea of objective and knowable laws (Pluckrose and Lindsay, 2020). Gergen (1973) in particular denied that social behavior is lawful. He argued that culture, time, and individual reactions against presumptive laws erode any predictable regularities researchers might wish to consider lawful. Indeed, Gergen claimed that in the social domain any regularities that might aspire to the status of law are self-nullifying. To overcome these difficulties, Gergen (2001) endeavored to reimagine social psychology along postmodern lines, but he did not provide clear guidance of what to do next (Krueger, 2002). Whether no attempts to explain human behavior can rise above the *narratological* or the *perfomative* only a postmodernist can know. I suspect, though, that most postmodernists would endorse the view that humans share a need to gain self-understanding. From this assumption it appears to follow that a credible story that makes sense of specific events or phenomena is a universal desideratum (Spoehr and Spoehr, 1994; Mukharji and Zeckhauser, 2020). In contrast, there is no obligation to shed light on what will happen next. At least in theory, humans have the option to assert – as Aischylos did – that the future is unknowable. With these caveats, I accept the sovereignty of experimental psychology and ask whether we can clarify the relationship between prediction and explanation. If we can, we might be able to mitigate the apparent irreconcilability between modern and postmodern psychology.

## The Future and the Past

Kierkegaard (1843) observed that “the philosophers say that life must be understood backwards. But they forget the other proposition, that it must be lived forwards.” The ordinary person, as it were, walks through life facing backward. Events and experiences come into view only when they begin to recede into the past. This person understands that looking forward and looking backward present unique challenges, and they may wonder how these two perspectives might be related to each other. Is, for example, a retrospective causal analysis and explanation necessary for making predictions about the future? Can predictions, if they turn out to be accurate, inform explanations of events that had occurred before the predictions were tested?

If there were no relationship between prediction and explanation, how might explanation be grounded? Perhaps explanations are worthwhile in their own right even if they cannot improve predictions. An appeal an explanation’s intrinsic value must steer clear of tautology. What is gained if all we can say that we like a good story because it is compelling? Perhaps a good story provides meaning, and the attainment of meaning can enhance subjective well-being (Baumeister, 2018). On this view, finding meaning is a pleasure not unlike the gratification of a physiological drive (Chater and Loewenstein, 2016). This pragmatic justification of the explanatory project requires more empirical study. Even if positive correlations between perceived meaning and subjective well-being are found (Ho et al., 2010), there are grounds for skepticism. Some explanations may be accepted uncritically simply because they are plausible. Many conspiracy theories provide no testable predictions, and the explanations they offer are factually false (Douglas et al., 2017). Yet, such theories can offer an illusory sense of meaning and understanding (Forgas and Baumeister, 2019).

Why would anyone seek valid predictions if these predictions do not contribute to an understanding of the past? To be sure, the search for successful predictions is valuable because instrumental rationality demands that choices depend on future prospects and not past outcomes (Dawes, 1988; Krueger, 2000). If predictions and their outcomes also improve an understanding of the past, this is an added benefit. There should be some convergence between prediction and explanation if there is any truth to the idea that Nature is lawful.

In a complex world, it is difficult to separate signals from noise. Prediction and explanation are hard, but they are hard for different reasons. Consider the reasons that might be offered for the claim that one is harder than the other. Advocates of prediction may note the intrinsic uncertainty of the future (Prigogine, 1997). Making predictions is risky, and risk and uncertainty are fundamentally aversive (Frenkel-Brunswik, 1949; Gigerenzer, 2014; Krueger and Grüning, in press; but see Hertwig and Engel, 2020, for significant exceptions). Data may refute the hypothesis. Errors are great teachers, but they inflict pain. Advocates of prediction may assert that past-oriented explanations are easier because the past has provided data that are now on the table, ready to be investigated. Investigators can look for probative evidence until they have reached a threshold at which they are willing to consider an explanation sufficient (Pennington and Hastie, 1992). A rewarding state of certainty is attained, and there is no immediate fear of failure.^4^ Finding an explanation is not a bet. The past is always there to be explained or reinterpreted, but the future continually wastes away and turns into the past. Once an event occurred, we cannot go back and predict it again.^5^

Conversely, advocates of explanation may note that their work is harder because of the complexities of causality. There are usually many potential causes to explain an event, affording many possible stories and interpretations. The explainer has to distinguish between necessary and sufficient causes, and decide how many of each should be part of the explanatory account (Kelley, 1972a, b). Explanation is hard because the potential causes can only be fitted to the data; they cannot be tested, which would require prediction and new data. Use of thought experiments with counterfactuals is one tool to evaluate the fidelity of a causal account, but then again, counterfactual causes and effects are only that – imaginary (Norton, 1996). The subjective satisfactoriness of a causal account is a poor guide to its validity.^6^ Someone might present a better account, but it is not clear what it would take for an account to fail in the kind of decisive way in which a prediction can fail.

In short, both advocates of prediction and advocates of explanation may claim that either approach is the harder one, depending on their epistemic or rhetorical aims. If there is a difference in difficulty, one wonders if it is an essential one or one that merely reflects differences in knowledge or differences in what advocates wish to emphasize. Russell (1913) once argued that there is no essential difference between predication and causation, and that these terms should be “extruded” from the philosophical vocabulary. Assuming perfect determinism in the tradition of Spinoza or Laplace, Russell argued that the direction of the flow of time has no bearing on how events are related to one another or how contingent they are on others. An omniscient being could wind the universe forward or backward, and the deterministic laws would reveal themselves in the same fashion. Requiring only the capacity of perception, this omniscient being would have no need to “think” in the way ordinary humans do when struggling to make a prediction or find an explanation. The need for prediction and explanation is a function of human ignorance. Of course, Russell’s hypothetical bird’s-eye view is a metaphysical *amuse bouche* (Prigogine, 1997). It should, however, remind the predictors and the explainers that they are looking at the same Nature, albeit from different angles.

Experimental psychology is dedicated to the study of causes and the “explanation of variance” with a forward-looking logic. Hypotheses are bets about data not yet seen. A potential cause is activated in the laboratory and its effects are observed. Yet, past-oriented explainers want a causal account of things that already happened. To the experimentalist, the question is this: Once a cause C is found to be sufficient to produce effect E, how can the presence of C be inferred once E has come into evidence? In other words, when predictors and explainers converse, they can take the problem of reverse inference as their common ground (Krueger, 2017b).

## Inferences in the Lab and in the Wild

By formalizing the relationships among unconditional and conditional probabilities, Bayes’s Theorem provides a framework for thinking about reverse inference. Before reviewing the theorem and some of its implications, consider a thought experiment to illustrate the divergent interests of predictors and explainers. An experimenter has proposed the following hypothesis: “Men who want power admire men who have power.” This sounds simple enough and perhaps tautological, but it is a prediction that might be worth testing. The experimenter measures the need for power in each of a number of sampled male participants. The experimenter then randomly assigns the participants to an experimental and a control condition. The men in the experimental condition receive a treatment – perhaps by way of priming or persuasion – designed to temporarily increase their need for power. The experimenter then measures the admiration these participants express for certain high- vs. low-power men.

Suppose that, as hypothesized, the experiment shows that the experimental manipulation did not affect admiration for low-powered men but did produce a strong effect on the admiration for high-powered men. Suppose for simplicity that the data are normally distributed within each condition and the difference between the two means is one standard deviation. The experimenter can now ask how probable it is that a randomly drawn participant had received the experimental treatment. In this symmetrical case, forward and reverse inferences yield the same result. The probability of a participant with a score above the grand average to belong to the treatment group is the same as the probability of a participant from the treatment group to have a score above the grand average. With *d* = 1.0, this probability is 0.69. An effect size of *d* = 0.4, which is empirically more realistic, yields a rather modest categorization benefit of *p* = 0.58, with *p* = 0.5 being the floor of ignorance.

This weakness of reverse inference for a typical effect size highlights a critical feature of experimentation: the narrow focus on group averages (Danziger, 1994). An individual’s score is modeled as the sum of the group average and an “error” term, which comprises both the imperfections of measurement and whatever it is that makes the individual unique (Lord, 1959). The laws pursued by experiments sampling participants do not exhaust all that Nature has to offer; they are limited to group trends. If, in the above example, the difference between the two group means were to remain the same while the variance of the individual’s scores increased within each condition, the standardized effect size would shrink and reverse inferences would become even more uncertain.

Inferences after experimentation tend to underestimate the true effect. In the hypothetical experiment, the need of power was measured first, but it was not used to predict the admiration for powerful men. Individual differences on this measure were treated as error variance. If there is is a correlation between need for power and admiration of the powerful within conditions, it seems likely that this correlation contains a causal effect of need on admiration. This situation is analogous to one where a correlation between “need” and “love” is observed in the wild. When an experiment shows that manipulated need predicts heightened love, this finding affords the inference that the original, non-experimental, correlation contains a causal path from need to admiration.^7^

Experimental results understate the difficulty of making causal inferences in the wild. In the wild, many potential causes remain in play. Whereas the skillful experimenter eliminates uninteresting causes *a priori*, the skillful explainer must be an expert explorer. The task is to detect and eliminate improbable causes after the fact. In a world of many causes and many effects, the scenarios faced by the predictor and by the explainer look different. The predictor is interested in one cause, C, realizing that it may have several effects, E_1_ to E_k_. Being interested in this one cause and in only one particular effect, say E_1_, the experimenter has attempted to manipulate only this one cause and to neutralize all others by randomly assigning the participants to the experimental and the control conditions; the other potential effects are dismissed by not being measured. The task is to see if C predicts E_1_. In contrast, the explainer has selected a particular event or effect E, and wants to know which of several potential causes, C_1_ to C_*k*_, is the most effective one. Where can the predictor and the explainer meet?

## Bayesian Reverse Inference

Bayes’s Theorem shows that the probability of a cause C given an effect E is equal to the product of the prior probability of C and the “diagnostic ratio,” which is the probability of the effect E given cause C over the unconditional probability of E, or

(1) $$\text{p}\,\left(\text{C}|\text{E}\right)=\text{p}\,\left(\text{C}\right)\cdot \frac{\text{p}\,\left(\text{E}|\text{C}\right)}{\text{p}\,\left(\text{E}\right)}$$

A one-to-one association between experimental results, which yield p(E|C), and what the explainer wants, which is p(C|E), is limited to the special case in which the cause is as probable as the effect *a priori*, that is, if p(C) = p(E). Such an equality is rare. The probabilities of C and E are often unknown, but there are regularities that provide common ground for predictors and explainers.

Consider first the implications of the prior probability of the effect. This probability is equal to the sum of the products of the unconditional probabilities of the various causes in play and their corresponding conditional probabilities of yielding the effect, or

(2) $$p\,\left(E\right)=p\,\left(C_{1}\right)\cdot p\,\left(E|C_{1}\right)+\ldots \,p\,\left(C_{k}\right)\cdot p\,\left(E|C_{k}\right)$$

To understand the implications of this equation, consider the case where all causes are ineffectual, that is, they neither promote nor inhibit the effect. All diagnostic ratios, that is, all p(E|C_i_)/p(E) = 1. There are three important implications. First, once one cause is found to have a diagnostic ratio >1, the diagnostic ratios of all other causes fall below 1. As one cause is identified as promoting the effect, all others must now be assumed to be inhibitors. Second, p(E) increases if at least one p(E|C_i_) > 1, while all other p(E|C_i_) remain the same. That is, the price of having found some relevant causal information is that the effect is less rare than formerly thought. Third, as more causes of the promoting kind emerge, they reduce the number of inhibitory causes, and p(E) increases further while the diagnostic ratio of each individual promoting cause becomes smaller. Likewise, the inverse conditional probability, p(C_i_|E), for each promoting cause also becomes smaller, although their sum increases.

Consider a numerical example. Causes C_1_ to C_4_ each have a prior probability of 0.25. The first cause is perfectly promoting, p(E|C_1_) = 1, while the other three are perfectly inhibiting, p(E|C_2 to 4_) = 0. Now, p(E) = 0.25, the diagnostic ratio is 4 for C_1_ and 0 for the three others, and the inverse conditional, p(C|E) is 1 for C_1_ and 0 for the three others. Next, we assume that C_1_, C_2_, and C_3_ are found to be maximally promoting, that is, p(E|C_1_) = p(E|C_2_) = p(E|C_3_) = 1. The result is that p(E) rises to 0.75, while the diagnostic ratios of the promoting causes fall to 1.33, and their probabilities given the effect fall to 0.333. For any particular number of presumed causes, the more frequent the effect is (the higher p(E) is), the less effective individual causes are. Frequent events are thus difficult to explain with a parsimonious account, that is, an account that requires few causes. The more promoting causes there are, the more common the effect is likely to be and the weaker is the role for each individual cause. By contrast, rare events are potentially well explained by few – perhaps even just one – highly effective causes.^8^ Conversely, common effects are easy to predict. One need only bet on common events of the past to repeat themselves (Hull et al., 1947; Ouellette and Wood, 1998). Often, the predictors of common effects are not even referred to as causes, but simply as “conditions,” or general states of nature prevailing before and after the appearance of the effect. In contrast, rare effects are difficult to predict (Taleb, 2007; but see Lindaas and Pettersen, 2016). Black swan events are infamous for not having been predicted but then having been explained *ad libitum*.

Now consider the consequences of variation in the prior probability of a cause. The more probable a cause is, the less likely it is to be highly effective. This is so because the cause’s effectiveness is captured by the diagnostic ratio of p(E|C_i_)/p(E) and because p(C_i_) is part of the denominator (see Eq. 2). A rare cause that explains an effect that would otherwise not occur is most attractive. A compelling explanatory account reveals how an unusual or extraordinary event came about thanks to the force of a single cause that would otherwise rarely be seen. Many of humankind’s legends and myths comprise rare causes stirring up extraordinary outcomes. Achilles gets mad only twice, when Agamemnon steals his concubine Briseis and when Hector kills his friend Patroclus. In both cases, Achilles responds promptly, with sullen retreat and mortal rage respectively after the theft and after the murder. Good stories are memorable because they provide a crisp causal account (Schank and Abelson, 1977). Vivid one-to-one cause-and-effect associations do not require laborious probability calculations; they are open to “direct perception” (Heider and Simmel, 1944), particularly if they obey Hume’s contiguity requirement in time and space. The explanatory causal account forces itself upon the observer. But had these events and their consequences been predictable? Our myths and legends are thick on causal stories, but thin on predictions. There is the occasional dark prophecy, which usually goes unheeded, or, as in Aischylos’s Prometheus, there is “blind hope.” Conjunctions of rare causes and rare events are the pleasures of the explanatory mind (a temptation that I will indulge in the second section of this article), but a challenge to those who seek to make successful predictions. Yet, many experimental psychologists wish to demonstrate causal relationships that seem unlikely at the outset for fear of having demonstrated what turns out to be trivial, tautological, or familiar in folk psychology (Felin et al., 2019). Researchers must “anticipate the unexpected,” and do so without making it seem paradoxical (Fiedler, 2017). The more experimentalists pursue the high-hanging fruits of risky hypotheses, the more often they will fail, thereby stoking the discipline’s replication crisis. But when they succeed, they may be able to make non-trivial contributions that are attractive to students of history (Krueger and Heck, 2018).

## History Inspires Research Which Helps to Explain History

I now turn to the question of whether reverse inferences from theory and experimental findings can help explain past behavioral episodes. Can psychological research shed light on events that are otherwise the reserve of historical analysis or folk psychological interpretation? Note that the question of whether such linkages can be found is different from the mission and scope of applied social psychology, which seeks to generalize interventions that have been found to work in the lab. Like basic theory-driven work, applied social psychology is concerned with the optimization of future outcomes, that is, with making predictions (Forgas et al., 2020).

As to the influence of the wild on the lab, the history of social psychology is instructive. Early social psychological research advanced in part by responding to social problems such as Anti-Semitism (Adorno et al., 1950; Martin, 2001), other ethnic stereotypes QQ (Katz and Braly, 1933; Krueger, 1996a, b), excessive conformity (Asch, 1956; Constant et al., 2016), or yielding to propaganda (Hovland et al., 1953; Osterhouse and Brock, 1970; Lewandowsky et al., 2013). Two iconic research programs owed their existence to specific historical events and the expectation that experimental results would shed light on why the actors behaved as they did. Arendt’s (1963) account of the Eichmann trial stimulated Milgram’s (1963) obedience studies. Arendt suggested that obedience to authority is a sufficient cause of abhorrent behavior, and is perhaps the preponderant cause. Milgram sought to show that the essential dynamics, that is, the lawful regularities, of authority and obedience can be reproduced in the laboratory. During that same decade, the murder of Kitty Genovese prompted Latané and Darley (1968) to experimentally reproduce the phenomenon of bystander apathy. These real-world events were striking and were considered improbable at the time; yet they could be demonstrated by experimental research. For the purposes of this article, it is worth noting that reverse inferences from the data could extend beyond the hypotheses tested in the laboratory to retrograde explanations of the historical events that had inspired the research. What is more, when there are new instances of violence in a state-sponsored context or failures to intervene on behalf of others in need, the findings of experimental psychology contribute to the construction of causal accounts.

Today, a problem-focused approach to research continues (Krueger and Funder, 2004) with comparatively little attention paid to applications, interventions, or historical analysis. Many researchers focus on theory development and theory testing, where applications or reverse inferences are left to others. Much progress has been made in theory development and theory testing, although it is not always clear what is meant by “theory.” Rigorous hypothetico-deductive processes are not the rule in experimental design (Prager et al., 2018), and many researchers settle for vague verbal descriptions of phenomena (Gigerenzer, 1998, 2010).

The three historical cases, or case studies, described below are meant to illustrate how research evidence obtained in the laboratory as well as theoretical analysis can inform historical case studies. Intended not as proofs of concept but rather as suggestions of concept, these vignettes show that theory-grounded research can make useful contributions to historical analysis. The first vignette seeks to improve the understanding of a man’s fateful personal decision not only with reference to the historical context but also in light of cognitive research on decision-making. The second vignette seeks to show how an extreme case of identity transformation may be understood in light of theory and research on escalating commitments. The third vignette uses a contemporary theory of strategic behavior in addition to experimental findings to reconstruct an interpersonal dynamic and its – as it turns out – benign resolution.

## Philipp Von Hutten: Making Fateful Decisions Under the Shadow of Death

During the first half of the 16th Century, German bankers financed several expeditions into what was then known as “Little Venice,” or Venezuela. The goal of these expeditions was to subjugate the native population and to exploit its natural and cultural resources. Little is remembered today about this chapter of American history. Hence, the travelog and the letters of Philip von Hutten are of great interest (Schmitt and von Hutten, 1996). Hutten’s activities and experiences can be seen through a politico-historical lens, with an emphasis on the European project to conquer and colonize the Americas. There is nothing in the present analysis to detract from this approach. The question of interest here is whether the available information sheds light on Hutten’s psychology, and whether contemporary research on judgment and decision making can help illuminate his fateful decision to carry on (Krueger, 2013).

The critical event is Hutten’s decision to mount a third expedition into the Venezuelan hinterland when two prior multi-year expeditions had already failed to bear fruit. Hutten was deep in debt, needing to replenish supplies without having made enough plunder to cover the costs, and it had become clear that the prospects of finding riches were remote. His family in Germany seems to have understood his dire circumstances. They offered to pay his debts if he returned home. Hutten refused, noting the unacceptability of the ridicule he was sure to suffer at the hands of his German neighbors. This is a psychological element of note, as it expresses the ethics of honor that was standard for men of Hutten’s caste (Cohen et al., 1996). In social psychological terms, Hutten was concerned with the image he would project and the judgments he would receive in the social world to which he would return (Krueger et al., 2020).

Is Hutten’s fear of being judged a sufficient explanation? Although such fears can be debilitating, few prefer death to ridicule. The record does not suggest that Hutten was suicidal. He was aware of the mortal risks he was facing, but he did not foresee that he would be captured and beheaded by a Spaniard who was a rival for the position of governor. The critical question is how he evaluated the chances of a third expedition to finally yield the badly needed rewards. A first pass is to submit that Hutten was overconfident in predicting success. Excessive confidence precipitates more failures than successes in life and business (Moore, 2020).

Beyond overconfidence lies the possibility of escalating commitments culminating in a sunk cost fallacy (Arkes and Blumer, 1985; Arkes and Ayton, 1999; Feldman and Wong, 2018). Commitments escalate as each successive investment of time, money, honor, or other material or psychological resources makes additional investments more likely regardless of real or imagined profits. Research on escalating commitments itself was inspired by famous failures in business and war. For example, the protraction of the Vietnam war beyond the point of its apparent failure presented a challenge to researchers to model, predict, and ultimately understand patently irrational behavior. Why, or under what conditions, are some individuals willing to do what they don’t want to do – and even when they have an exit option? Why, in economic terms, would people pay for something they are loath to do?

Hutten’s own documents show that he knew his previous expeditions were failures, that he knew his planned expedition had a low expected value of success (although it cannot be proven that he thought this value was negative), and that he knew that his family was ready to cover his debts. In reconstruction, the confluence of the ethic of honor along with the pull of sunk costs as documented by experimental research, provide a sufficiently plausible explanation for his fateful decision to persist (Krueger, 2013)

## Gonzalo Guerrero: Transforming Identity One Step at a Time

A few years before Hutten, another European adventurer’s life took an unexpected turn. Gonzalo, also known by the honorific appellation “Guerrero,” was not a nobleman or would-be conquistador, but a mariner sailing with a Spanish expedition to Panama. The attempt to build a colony there failed. The Spaniards decided to return to Cuba, but their ship sank off the coast of the Yucatán. The survivors were captured by local Maya who proceeded to eat all but two of them. Gonzalo and a padre named of Jerónimo de Águilar were spared to be consumed later. The two escaped and found refuge with a rival tribe, were enslaved again, but evaded mortal threat. This is the beginning of a legend as told by the chroniclers (de Landa, 1566) and contemporary Gonzalo scholars (Calder, 2017). Their common source was a single eyewitness, namely Águilar. Perhaps Gonzalo never existed. His story could be a myth. For the present project, this does not matter. His story raises questions about what humans are capable of doing *in extremis*, and whether experimental psychology can help explain how.

Gonzalo not only survived but flourished. In time, he won his captors’ trust and respect, married a chief’s daughter, begot children, and became an influential war captain. Along the way, he became more Maya in his thinking, feeling, and acting. The outward signs of his transformation are critical. In the Yucatán today, where he is revered as an ancestral figure, he is depicted with tattoos, piercings, and various native ornamentations. Yet, statues and paintings also show him with a beard to note his origins (Mueller, 2001).

If Hutten escalated his commitments toward a fateful business and life decision, Gonzalo went down a road of stepwise identity transformation (Krueger, 2017a). By what he did, he changed who he was. It is not clear whether Gonzalo’s transformation comprised sunk costs. A demonstration of sunk costs requires evidence of costs outpacing rewards, while stopping remains possible. Gonzalo himself, however, claimed that his life had improved. According to Águilar, he declined to rejoin the Spaniards when given the chance. Pointing to his family and the social status he had achieved, Gonzalo asked Águilar if it made sense for him to forsake it all. The life he had built was a rewarding one, and his decision to cultivate it further might well have been rational.

Although the Gonzalo legend shares with Hutten’s story the mechanisms of escalating commitments (Burger, 1999; Krueger and Massey, 2009), it shows how – under the right circumstances – such commitments can yield extraordinary results. As a thought experiment, the Gonzalo legend explores the limits of identity transformation, a process millions of immigrants and other travelers confront every day (Hong et al., 2016). By asking what is possible, Gonzalo poses a special challenge to experimental research staked on the prediction of averages in the search for general laws.^9^

One line of empirical research that can contribute to an understanding of the Gonzalo experience is work on world-class performance. The late K. Anders Ericsson showed in a multi-decade research program that sustained practice at the edge of one’s ability eventually yields the rewards of world-class performance (Ericsson, 2016). The effects of sustained and challenging practice on the attainment of expertise were first demonstrated in narrowly defined performance domains such as playing chess or playing a musical instrument (Ericsson et al., 1993). The constraints of a narrowly defined space of performance are crucially important. Loosely or ill-defined domains such as “leadership ability” or “wisdom of life” do not allow the model to work (Krueger, 2020).

A person’s project to assimilate into a new culture entails a broader set of skills than playing chess, but it is still more clearly defined than “being wise.” Hence, Ericsson’s theory of expertise applies *mutatis mutandis*. The mastery of a formerly alien culture requires the acquisition of a new language, a code of conduct, an understanding of norms and expectations, along with the acquisition of other forms of “tacit knowledge” (Polanyi, 1966). The dedicated immigrant is drawn into multiple correlated expertise projects, which can be fueled further by irreversible commitments (Schelling, 1956; Koziel et al., 2010). In short, Gonzalo’s life is beginning to make sense in light of theory-grounded psychological science – without losing its charm.^10^

## Robinson Crusoe: Finding Social Peace But No Equilibrium

In his masterly novel, Defoe (1719/1998) explored a man’s response to extraordinary circumstances. Shipwrecked, Robinson Crusoe solves the problems of survival without human company, only to discover that the presence of other humans brings new challenges, which are in some ways more daunting. Driven more by fear and prudence than by aggression or greed, Crusoe kills a party of cannibals, and saves the life of one of their victims. He names him Friday, as it was on a Friday that he found him. Crusoe is an even poorer fit to the prototype of the conquistador than are Hutten or Gonzalo. Yet, Crusoe has guns, while Friday has none; and Crusoe saved Friday’s life, although he can’t be sure whether Friday will remember the deed with gratitude.

The psychological questions are “How did Crusoe and Friday manage to get along?” and “Why did they not kill each other?” Some social scientists have recognized the intellectual and theoretical appeal of this puzzle (Tsebelis, 1989; van Lange et al., 2014), but have presented no analysis. I attempted one (Krueger, 2014a, b), which I summarize here. This analysis is theory-driven and it can be enriched with experimental results.

The theory is the heterodox game “theory of moves” (Brams, 1993, 2011). This theory asks the analyst to generate a plausible rank ordering of the four outcomes pertaining to each interactant. These payoffs arise from the crossing of the two strategies each player has: to be aggressive or to be conciliatory. Relying on my reading of Defoe’s account, I suggest the following rank order for Crusoe. Crusoe’s primary interest is to have a conciliatory (i.e., submissive) Friday, and if Friday submits, Crusoe is happy to be conciliatory as well. If, however, Friday is aggressive (i.e., rebellious), Crusoe would rather fight than flee. Friday, for his part, is primarily interested in Crusoe – who has guns – to be conciliatory. But if Crusoe is indeed gentle, Friday has an incentive to rebel. Otherwise, Friday will submit to an aggressive Crusoe (again, Crusoe has guns).

**FIGURE 1:**
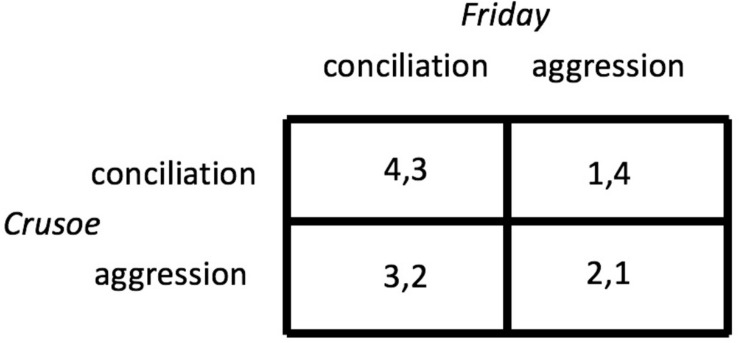
The power game in matrix form. The row player’s (Crusoe’s) payoffs are shown to the left of the comma. Higher numbers are better.

With the two sets of preferences in place (shown in Figure 1), a game theoretic analysis looks for a player’s best response assuming the other player’s preferences are known. Both Crusoe and Friday face a tricky dilemma because neither has a dominating strategy and the game has no unique Nash equilibrium.^11^ The best collective outcome is obtained if both players are conciliatory, and this is how Defoe tells it. This leaves Crusoe with his best (4) and Friday with his second-best outcome (3). This may seem unfair, but given the player’s misaligned preferences all joint outcomes are unfair.

The theory of moves raises interesting questions, two of which I will address. The first question is whether Crusoe is more powerful than Friday, and if so, how this advantage is encoded in the preference rankings. Crusoe’s doing better than Friday is an indication of his greater power, but we cannot consider it sufficient without courting an outcome bias. Crusoe’s advantage in outcome is no clear signal of his greater power causing this favorable result. Crusoe’s and Friday’s primary interests are not diagnostic either. Both prefer the other to be conciliatory. Their secondary interests are different, however. Playing tit-for-tat, Crusoe claims the power to reward conciliation and to punish rebellion. Friday does the opposite. He would exploit conciliation and yield to aggression. If Friday also played tit-for-tat, the game would be a “stag hunt,” where mutual cooperation is easily achieved (Krueger et al., 2020). In other words, it is Friday’s own interest in power that makes Crusoe more powerful.

The second question is how a state of mutual conciliation can be maintained if it is not a Nash equilibrium. Here we see that it depends on Friday. Friday has an incentive to rebel, moving the game to “Crusoe 1: Friday 4.” Yet, Crusoe would counter by putting the rebellion down, yielding “Crusoe 2: Friday 1.” An intelligent player can foresee how this game would deteriorate into a cycle of war and truce, leaving average outcomes to both players that are inferior to the ones obtained with mutual conciliation. Viewed from this perspective, Friday is the wiser player and perhaps the more powerful one. He controls the keeping of the peace. The price he pays is the cost of his deference to Crusoe.

Findings from experimental social psychology and behavioral economics offer further insights. Social psychological research supports the notion that humans are sensitive to hierarchy and reluctant to challenge power when such challenges are risky and costly (Koski et al., 2015). Once established, challenges to power hierarchies have a better chance of succeeding if the challengers form alliances (DeScioli and Kurzban, 2009). Single challengers are at the greatest risk of failing. A behavioral economics perspective notes the similarity between the final outcome of “Crusoe 4: Friday 3” and the typical result of an ultimatum game (Güth, 1995). Crusoe claims his first preference and asks Friday to settle for something short of his, Friday’s, maximum. Most participants in ultimatum games accept such a positive if mildly unequal distribution. Taken together, the present *post hoc* analysis of Defoe’s fiction shows that to the psychologist, Friday is the more interesting character.^12^ Some postmodernists will agree, and one author re-told the Crusoe story from Friday’s perspective. In his account, “*Vendredi*” introduces Crusoe to the superior ways of living untarnished by the European Enlightenment (Tournier, 1969). Supposing that one fiction cannot disprove another, I settle for showing that psychological science can help explain behavior in rational terms.^13^

## Discussion

My attempt to make theoretical and experimental (social) psychology useful for the interpretation of observed (or imagined) behavior is only a sketch. There is no smooth path from theory and experiment to a sufficient explanation of historical (or fictional) events. In the first section of this article, I reviewed the logic and the challenges of reverse inference in rather abstract fashion. In the second section, I illustrated the potential of theory and research to help explain real and imagined historic events. To conclude, I discuss three issues that complicate this enterprise, although I do think that the challenges are surmountable. I begin with the question of whether behavior, in order to be explained, must be “real.” Then I ask whether, as a reverse inference, a causal account of behavior is different from other types of category judgment. Finally, I explore to what extent outcome bias can affect both predictions and explanations.

## Fiction, What Fiction?

Moving from Hutten to Gonzalo to Crusoe, we descended from reasonably well-documented history to legend to pure fiction. I have suggested that the methods of causal analysis apply regardless of where the events of interest are located on this spectrum. How might this claim be justified? One response is that no such justification is possible. On this view, real and imagined behavioral episodes are different kinds. They differ from one another much like lived experience differs from dreams. If so, it is dangerous to ignore this difference in kind and to treat it as if it were a matter of degree. Philosopher Nozick (1981) argued that people, as a rule, prefer fact to fiction, no matter how pleasing this fiction might be. Ironically, Nozick made this point by way of a thought experiment. A thought experiment is fiction, and fiction can simulate and explain reality by bringing into focus critical similarities and differences. All counterfactuals are *per definitionem* fiction, but they are useful in causal analysis (Byrne, 2016). This assumption allows the factual and the counterfactual to switch roles. If the counterfactual can help explain the factual, so can the factual help explain the counterfactual.

There are parallels between lived events (“factual behavior”) and imagined events (“fictive behavior”). Both are entangled in the causal web of the world. Fiction that violates the constraints of this web strikes us as bizarre or entertaining as “science” fiction. Fiction becomes “relatable” inasmuch as it enables readers to construct a causal story that makes sense of what happens. Doing so, they perform the same cognitive operations they would perform for real events. A causal analysis of a piece of fiction, if it is informed by findings obtained with future-oriented, that is, predictive, psychological science, is comparable to a causal analysis of a fact. Both analyses are simulations,^14^ whose goal it is to provide a satisfactory explanatory account, or a “story,” with an acceptable goodness of fit. Such an account cannot be refuted in the same way that a prediction can be refuted by data. A good causal story can only be replaced by a better one, if such a story comes along.

## Causes as Categories

I have argued that future-oriented and prediction-based science can aid past-oriented and explanation-based scholarship and lay cognition. Bayes’s Theorem served as an orienting framework. Inferences from the future to the past are reverse inferences. They tend to work, but without more information or strong assumptions, it is difficult to know just how well they work; one only knows that making such inferences works better than doing nothing (Krueger and Heck, 2017).

A skeptical view is that there is little that is new because the benefits and limitations of reverse inference are well known (Dawes, 1988; Krueger, 2017b). An implication of this sort of skepticism is that causal inference reduces to category inference and that therefore the cognitive errors corrupting the latter also corrupt the former. This claim has merit inasmuch as one is willing to assume that anything that can be modeled with Bayes’s Theorem is of the same kind. In psychology (Kahneman and Tversky, 1973) and in medical science (Eddy, 1982), processes of category judgment have attracted a great deal of attention (Fiedler and Grüning, in press). The signature finding is the so-called base-rate fallacy, which occurs when people place an instance into a small category if the probability of that instance is high *given* membership in that category (see Gigerenzer and Hoffrage, 1995, or Koehler, 1996, for critical evaluations). The base-rate fallacy is a one-to-one reverse inference that ignores the prior probability of the category. In the medical context, this fallacy entails overdiagnoses of rare diseases. Even many trained diagnosticians confuse a test’s sensitivity with its positive predictive value (Franklin and Krueger, 2003). People, to paraphrase Robyn Dawes, assume a symmetry Nature does not provide (see Krueger, 1996b, for other violations of Bayesian inference).

From a Bayesian perspective, one might assert that causes are categories just like other categories. This view can be psychologized by saying that once a sufficient cause has been found for the event of interest, this event is placed into the category comprising all those events that result from this particular cause. The usual psychological biases would occur, and the base rate fallacy is of particular concern. The fallacy would be present if, after observing a high p(E|C), the perceiver concluded that p(C|E) is also high even if p(C) is low. In other words, the behavior would be attributed to a potent cause too rare to emerge as the most probable.

If causes were just a kind of category, the work of inference would be simple. Arguably, however, there is a difference. A complete causal inference has two elements. The identification of a probable cause where the resulting p(C|E) passes some threshold, say 0.5, is only the first element. The second element is the identification of a causal process. In Bayes’s Theorem, this process is represented by the extent of belief updating, which is captured by various ratios or difference scores [e.g., p(E|C)/p(E) or p(C|E) – p(C)]. The most convincing causal claims comprise both a causal categorization where p(C|E) is high and a causal process where p(C|E) – p(C) is large. The two elements are conceptually independent, though statistically related (Krueger and Heck, 2017). It is the latter element, the causal process, that gives energy to the human tendency to convey lessons of causality in story form.^15^

## Outcome Bias in Inferences About the Future and the Past

Along with overconfidence, confirmation bias, innumeracy, and downright foolishness, outcome bias is one of the signature threats to rational reasoning. Baron and Hershey (1988) made the canonical case, and the bias has been documented many times since. The bias occurs when the outcome of a decision contaminates evaluations of decision quality. When a decision maker takes a calculated risk or makes a decision under uncertainty, the evaluator should limit the assessment of decision quality to the information the decision maker had (or failed to obtain) and to the processing of this information. Information the decision maker did not have or could not have at decision time ought to be excluded from the assessment. The outcome of a decision, by definition, follows a decision, and must therefore be ignored (Krueger and Acevedo, 2007). A lottery winner may be congratulated for having had good luck but should not be praised for having the gift of prophecy; a loser should not be blamed for losing. If anything, both should be blamed for playing in the first place as lotteries have steeply negative expected values.

The concept of outcome bias straddles the tasks of prediction and explanation. The decision maker is engaged in a prediction task, and the observer evaluates how well this task is performed. To do this, the evaluator simulates the process of making the prediction. Outcome bias occurs if the evaluator bestows too much praise on the decision maker after a positive outcome, or too much blame after a negative outcome (as noted in the lottery example above). At this level, outcome bias is unrelated to the question of *why* the decision makers made a particular prediction.

How might outcome bias affect explanations and causal inferences? The booming field of moral psychology has dedicated itself to the question of how people assign blame to transgressors. It was once believed that an outcome bias exists such that the weight of a negative outcome directly predicts the degree of assigned blame regardless of other considerations such as the transgressor’s mental state at the time (Mazzocco et al., 2004; Cushman, 2008). This view is no longer viable (see Malle et al., 2014, for an extended review and discussion). Malle et al.’s (2014) “theory of blame” stresses the role of mental state inferences and especially inferences about intentionality. Intentional actions tend to be supported or justified by reasons, which in turn predict judgments of blame. Crucially, this theory and others in the field of moral psychology take the presence (or absence) of personal causation as a starting point to get to judgments of blame. These theories are not concerned with the question of what caused the person to act beyond the intention immediately preceding the act. A more comprehensive causal explanation would also address the origins of intentions. If we were to say that Hutten planned a third expedition because he wanted to, we would be making a circular argument (Greve, 2001); likewise for the question of why Gonzalo became a Maya, or for the question of why Friday did not rebel against Crusoe.

It would be a severe case of outcome bias to infer intentions only from outcomes. Early attribution theorists (Heider, 1958; Jones and Davis, 1965) warned against this heuristic. In the present analysis of the three historico-literary cases, I took the actors’ intentions for granted, and asked about their underlying causes. It remains possible that outcome biases compromise such causal inferences. One might wonder, for example, if Hutten’s tragic end at the hands of a murderer influenced my conclusion that his decision to undertake a third expedition was rationally flawed. It should not.

## Conclusion

In the spirit of the arguments presented in this article I should ask “Why did I write this article?” There are several coincidental factors. The first and central of these factors is a longstanding concern with the question of how, as an experimental social psychologist, I can respond to those who ask how my discipline enables us to explain people’s behavior. To me, the question became “whether” my discipline produces answers. Recognizing the difficulty of the problem, coupled with the general apathy of the field with regard to this question, I experienced cognitive dissonance (Festinger, 1957). Finding myself unable to ignore the question and thinking I have learned enough to attempt an answer, I embarked on this writing project. In the spirit of looking for theoretically and empirically grounded explanations, I submit that my work has been motivated, at least in part, by a wish to justify my career-long investments in my profession (Alicke and Sedikides, 2011).

A second factor is a growing involvement with other disciplines, especially the humanities. The discussions of Hutten, Gonzalo, and the Crusoe-Friday team grew out of presentations given at international conferences dedicated to the legacy of Alexander von Humboldt, the champion of the universality and interconnectedness of all science. Each of the three stories has its own history and logic of having been selected at the time. Once selected, these stories were highly accessible to me. No claim of their representativeness is made. Relatedly, I discovered Steven Brams’s “theory of moves” when studying “scripture,” a form of literature. It was a perspective-shifting experience to see Brams (1980) apply his variant of game theory to the interpretation of myths and stories lying at the foundation of our civilization. That this could be done was, as it were, a revelation. Integrating the use of Brams’s tools with social psychological theory and research to explain historical or literary events then came naturally. Again, in cognitive-psychological terms, these tools had become chronically accessible (Higgins, 1996).

A final question is whether the present effort can make a contribution to the further development of psychological science. Satisfying the folk by presenting compelling explanations of past behavior is one thing; improving theory and practice is another. The present article contains no new normative recommendations but an invitation to see that the possible uses of social psychology have been underestimated.

## Data Availability Statement

The original contributions presented in the study are included in the article/supplementary material, further inquiries can be directed to the corresponding author.

## Author Contributions

The author did all the work on this article. Literature review, conceptualization, writing, preparation of the figure.

## Conflict of Interest

The authors declare that the research was conducted in the absence of any commercial or financial relationships that could be construed as a potential conflict of interest.

## Footnotes

1 Tellingly, Copernicans are not known as “Postptolemaians.”

2 See Busemeyer et al. (2011) for post-mechanistic foundations of cognitive psychology.

3 Cummins (2000) argues that psychology has had greater success predicting regularities in human performance in experiments than explaining this performance in lawful terms. Many regularities, as for example, those summarized by psycho-physical laws, are descriptive instead of explanatory.

4 Although perhaps there should be because of the risk of “explanation bias” (Mukharji and Zeckhauser, 2020).

5 An additional difficulty of prediction is that it requires the prediction of the causes of events in order to predict the events themselves.

6 Bowers and Davis (2012) present a pointed critique of “just-so stories” in cognitive science, and Bayesian theories in particular.

7 In trivariate correlational models, correlations between presumed mediators and criterion variables are easily mistaken as support for a causal claim (Fiedler et al., 2011), a claim that can, however, be corroborated by experiments manipulating the mediator (Spencer et al., 2005).

8 This Bayesian analysis is limited to sufficient causes, which are assumed to be mutually exclusive and exhaustive. Interactions among them are not being considered.

9 Hirschman (2013) noted that, compared with the physical sciences, the social sciences remain sensitive to the exploration and extreme and unique cases, an orientation he calls “possibilism.”

10 It is well to remember that Jerómino de Águilar outlived Gonzalo without taking the path of full acculturation. Águilar’s success suggests that Gonzalo had some choice in the matter, as an alternative survival strategy was available.

11 A strategy (aggression or conciliation) is dominating if it is the best response irrespective of the other player’s move. A unique Nash equilibrium is a combination of strategies that leaves neither player with an incentive to act differently.

12 Arguably, the title of the essay “How Robinson Crusoe managed his Man Friday” (Krueger, 2014a) was a misnomer. Friday’s strategic choices, it turns out, were more pivotal for the preservation of collectively beneficial outcomes. Friday, in other words, managed Crusoe.

13 Even if the *explanandum* itself turns out to be irrational – as in the Hutten case.

14 Whether performed on fact or fiction, causal accounts are simulations in the sense that causation is, in either case, a psychological moment added to represent the relationship of what is then called the cause and the effect (De Pierris and Friedman, 2018).

15 With regard to usage, I note that many Bayesians refer to inferences about categories as “predictions.” Indeed, the conditional probability of p(C|E) is also known as the “positive predictive value.” In contrast, I see causal inferences in the temporal context of judgments about the past, reserving the term “prediction” for inferences about events that have not yet occurred.
